# A population pharmacokinetic model of piperaquine in pregnant and non-pregnant women with uncomplicated *Plasmodium falciparum* malaria in Sudan

**DOI:** 10.1186/1475-2875-11-398

**Published:** 2012-11-29

**Authors:** Richard M Hoglund, Ishag Adam, Warunee Hanpithakpong, Michael Ashton, Niklas Lindegardh, Nicholas PJ Day, Nicholas J White, Francois Nosten, Joel Tarning

**Affiliations:** 1 Unit for Pharmacokinetics and Drug Metabolism, Department of Pharmacology, University of Gothenburg, Gothenburg, Sweden; 2 Faculty of Medicine, University of Khartoum, Khartoum, Sudan; 3 Mahidol-Oxford Tropical Medicine Research Unit, Faculty of Tropical Medicine, Mahidol University, 60th Anniversary Chalermprakiat Building, 420/6 Rajvithi Road, Bangkok, 10400, Thailand; 4 Centre for Tropical Medicine, Department of Clinical Medicine, Churchill Hospital, Oxford, UK; 5 Shoklo Malaria Research Unit, Mae Sot, Thailand

**Keywords:** Malaria, Piperaquine, Pregnancy, Population pharmacokinetics, Nonlinear mixed-effects modelling

## Abstract

**Background:**

Pregnancy is associated with an increased risk of developing a malaria infection and a higher risk of developing severe malaria. The pharmacokinetic properties of many anti-malarials are also altered during pregnancy, often resulting in a decreased drug exposure. Piperaquine is a promising anti-malarial partner drug used in a fixed-dose combination with dihydroartemisinin. The aim of this study was to investigate the population pharmacokinetics of piperaquine in pregnant and non-pregnant Sudanese women with uncomplicated *Plasmodium falciparum* malaria.

**Method:**

Symptomatic patients received a standard dose regimen of the fixed dose oral piperaquine-dihydroartemisinin combination treatment. Densely sampled plasma aliquots were collected and analysed using a previously described LC-MS/MS method. Data from 12 pregnant and 12 non-pregnant women were analysed using nonlinear mixed-effects modelling. A Monte Carlo Mapped Power (MCMP) analysis was conducted based on a previously published study to evaluate the power of detecting covariates in this relatively small study.

**Results:**

A three-compartment disposition model with a transit-absorption model described the observed data well. Body weight was added as an allometric function on all clearance and volume parameters. A statistically significant decrease in estimated terminal piperaquine half-life in pregnant compared with non-pregnant women was found, but there were no differences in post-hoc estimates of total piperaquine exposure. The MCMP analysis indicated a minimum of 13 pregnant and 13 non-pregnant women were required to identify pregnancy as a covariate on relevant pharmacokinetic parameters (80% power and p=0.05). Pregnancy was, therefore, evaluated as a categorical and continuous covariate (i.e. estimate gestational age) in a full covariate approach. Using this approach pregnancy was not associated with any major change in piperaquine elimination clearance. However, a trend of increasing elimination clearance with increasing gestational age could be seen.

**Conclusions:**

The population pharmacokinetic properties of piperaquine were well described by a three-compartment disposition model in pregnant and non-pregnant women with uncomplicated malaria. The modelling approach showed no major difference in piperaquine exposure between the two groups and data presented here do not warrant a dose adjustment in pregnancy in this vulnerable population.

## Background

Malaria is one of the most important infectious diseases with an estimated 216 million people infected worldwide in 2010 [1]. Pregnant women are at an increased risk of developing a malaria infection [2] and are at a higher risk of progressing to severe malaria [3-5]. *Plasmodium falciparum* malaria is a major contributor to maternal mortality in Sudan; around 37% of all maternal deaths between 1985 and 1999 at the Medani Teaching Hospital in Medani City, Sudan, were attributed to malaria [6]. Malaria also has severe effects on the foetus causing both foetal loss and low birth weight.

Artemisinin-based combination therapy (ACT) is recommended as first-line treatment for *P*. *falciparum* malaria in all endemic areas. The artemisinin derivatives have a very rapid parasiticidal effect, which substantially reduces the parasite biomass during the first days of treatment. These drugs have a short terminal elimination half-life and are, therefore, used in combination with longer acting anti-malarials, with the aim of preventing recrudescence by killing residual parasites. Combination therapies consisting of drugs with different mechanisms of action also reduce the risk of the development of drug resistance [7,8].

The oral fixed-dose combination of dihydroartemisinin and piperaquine has shown excellent efficacy in the treatment of *P*. *falciparum* malaria [9-13]. Piperaquine is highly bound to plasma proteins (>99.9%), has a large apparent volume of distribution, (103–874 L/kg), a low apparent elimination clearance (0.6-1.3 L/h/kg) and, therefore, a long terminal elimination half-life (12–28 days) [14-20].

Pregnancy has considerable effects on the pharmacokinetic properties of many drugs. Renal elimination, expression of metabolising enzymes, volume of body water and the degree of plasma protein binding all change during pregnancy [21-23]. This could result in lower drug plasma concentrations [24]. Previously published studies have reported a decrease in drug exposure during the later stages of pregnancy for artesunate, artemether, dihydroartemisinin, lumefantrine, sulphadoxine, atovaquone, proguanil and cycloguanil [19,25-31]. Other anti-malarial drugs (e.g. quinine and amodiaquine/desethylamodiaquine) show no differences in pharmacokinetic properties in pregnant women compared to non-pregnant women [32-34].

Only one previous study has investigated the impact of pregnancy on the pharmacokinetic properties of piperaquine in patients with uncomplicated *P*. *falciparum* malaria [19]. Pregnancy was found to affect the elimination clearance and the bioavailability of piperaquine, but with no change in total drug exposure. This was further supported by a non-compartmental analysis of the same study [35]. No published information is available on the population pharmacokinetic properties of piperaquine in pregnant or non-pregnant women in an African country.

The aim of this study was to describe the population pharmacokinetic properties of piperaquine in pregnant and non-pregnant women with uncomplicated *P*. *falciparum* malaria in Sudan.

## Methods

### Study design

The study was conducted at the New Halfa Teaching Hospital in New Halfa, Sudan. Clinical details and non-compartmental analysis results are reported in full elsewhere [36]. The participating women received a written and oral explanation of the study in their own language. If the woman could not read, the explanation was read to her. Ethics approval for the study was given by the College of Medical and Technical Studies, Khartoum, Sudan.

Symptomatic pregnant women with uncomplicated *P*. *falciparum* malaria in their second or third trimester attending the antenatal clinic in New Halfa were eligible to participate in the study. Non-pregnant women with uncomplicated *P*. *falciparum* malaria were also recruited as controls.

### Drug regimen

All patients received dihydroartemisinin-piperaquine tetra-phosphate (Duo Cotecxin, 40 mg/320 mg tablets, Beijing Holley-Cotec Pharmaceuticals, Co., Ltd.) once daily for three days. Drug administration was directly observed and taken with a glass of water under fasting conditions. The number of tablets was based on the patient’s body weight to achieve a daily dose of 20 mg piperaquine tetra-phosphate/kg.

Blood samples were obtained by venous puncture or a three-way tap. PCR, haematology and biochemistry samples were drawn before the first dose and on day 14. Blood samples (2 mL) for pharmacokinetic analysis were drawn pre-dose and at 1.5, 4, 8, 24, 25.5, 28, 32, 48, 49, 50, 52, 56, 60, 72 h after the first dose and on days 5, 7, 14, 21, 28, 35, 42, 49, 56, 63 and 90. The actual time of dosing and sampling were noted and used in the pharmacokinetic analysis. Blood samples were centrifuged at 2000×g for 10 minutes and plasma samples stored in liquid nitrogen until the samples were transferred to Khartoum there they were stored in −80°C.

### Drug analysis

The chemical analysis was performed using a previously published method with separation and quantification by liquid chromatography (LC) and tandem mass spectrometry (MS/MS) detection [37]. The LC-system was an Agilent 1200 system consisting of a binary LC pump, a vacuum degasser, autosampler and a column compartment. The MS-system was an API 5000 triple/quadruple mass spectrometer with a Turbo V ionization source. The lower limit of quantification (LLOQ) was set to 1.5 ng/mL and the lower limit of detection (LLOD) was set to 0.38 ng/mL. This method reported an intra- and inter-day precision of below 10% for all quality control samples.

### Pharmacokinetic and statistical analysis

The data were analysed using nonlinear mixed-effects modelling as implemented in NONMEM version VI (Icon Development Solutions, Ellicott City, Maryland, USA) [38]. Piperaquine plasma concentrations were transformed into their natural logarithms to increase the stability of the numeric analysis. Models were fitted to the data using the first-order conditional estimation (FOCE) method with interaction [39-41]. Census, version 1.1 [42], and Xpose, version 4.04 [43], library for R was used for model diagnostics. Perl-speaks-NONMEM (PsN), version 3.4.2, [44] was used to automate the modelling process and for model diagnostics.

Model discrimination was based the on the objective function value (OFV) computed by NONMEM as −2×log likelihood [45]. The OFV is approximately χ^2^ distributed and a decrease in OFV of 3.84 and 6.64 is considered a significant drop with p<0.05 and p<0.01, respectively, when adding one additional parameter (one degree of freedom between two nested models).

Structural models with one-, two-, three- and four- disposition compartments were fitted to the data. Several alternative absorption models were investigated; first-order absorption, first-order absorption with lag time, zero-order absorption, sequential zero- and first-order absorption, sequential zero- and first-order absorption with lag time, parallel first- and zero-order absorption, parallel first-order absorption and transit compartment absorption with a fixed number of 1–10 transit compartments. The full implementation of the transit compartment absorption model, which allows the number of transit compartments to vary between patients, was also evaluated [46].

It was assumed that drug elimination took place from the central compartment and the base model was parameterized as elimination clearance, central volume of distribution, inter-compartmental clearance(s) and peripheral distribution volume(s). Bioavailability was added to the model and the population value was fixed to 100%.

The distribution of the individual parameters was assumed to be log-normal and between-subject variability (BSV) was investigated on all parameters as an exponential random effect [Equation 1].

(1) $$P_{i}=\theta_{p}\cdot e^{\eta_{i,P}}$$

where *P*_*i*_ is the individual estimate for a model parameter (e.g. individual drug clearance) in the *i*^*th*^ individual. *θ*_*p*_ is the population mean of parameter *P* and *η*_*i*,*P*_ is individual *i*^th^ deviation from the population mean. BSV is estimated from a normal distribution with variance ω^2^ and zero mean. Between dose occasion variability (BOV) was evaluated on absorption parameters [Equation 2].

(2) $$P_{\mathrm{ij}}=\theta_{P}\cdot e^{\left(\eta_{i,P}+\kappa_{j,P}\right)}$$

Where P_ij_ is the individual parameter estimate for the i^th^ patient on the j^th^ dose occasion, κ is the deviation from the population mean after each dose occasion, taken from a normal distribution with variance Π^2^ and zero mean. An additive residual error model was assumed since data were transformed into their natural logarithms (i.e. essentially equivalent to an exponential error model on an arithmetic scale). Body weight was tried in the model as a simultaneous incorporation of an allometric function on all clearance (power of 0.75) and volume parameters (power of 1), considering the strong biological prior of this covariate relationship [47-49].

Basic goodness-of-fit plots and simulation-based diagnostics were used to evaluate the final model. Visual and numerical predictive checks [50] were performed using 2000 simulations at each concentration-time point with binning based on protocol times. The 5^th^, 50^th^ and 95^th^ percentile of observed data were plotted over the simulated 95% confidence interval of the same percentiles to evaluate the models predictive performance. Bootstrap diagnostics were performed using 1000 re-sampled datasets, stratified on pregnancy.

A Monte Carlo Mapped Power analysis (MCMP) [51] was conducted based on results from a previously published population analysis [19]. The current study design in terms of sampling times and doses were used to create a modelled data set with 408 pregnant and 408 non-pregnant patients. This data set and the published population pharmacokinetic model was used to estimate the minimum number of individuals needed in each group (i.e. pregnant and non-pregnant) to identify the described covariate relationships (i.e. a 45.0% increase in elimination clearance or a 46.8% increase in bioavailability) at a given power (80%) and significance level (p=0.05). Full power curves were produced by plotting number of patients needed against the power to detect the assumed covariate relationship.

Pregnancy was investigated in the final pharmacokinetic model utilizing a full covariate approach where pregnancy was included as a categorical covariate on all pharmacokinetic parameters. Estimated gestational age (EGA) was evaluated as a continuous covariate on individual parameters using a linear and a power function, and the most appropriate covariate relationship (lowest OFV) was incorporated into a full covariate model for EGA. These two full covariate models were bootstrapped (n=200) to investigate the impact of pregnancy. A pregnancy related change in the parameter estimate of more than 20% was deemed to have clinical relevance.

## Results

Fourteen non-pregnant and twelve pregnant women were recruited into the study but two non-pregnant women withdrew their consent and were excluded from the analysis. Full demographics are given in Table 1. The treatment was well-tolerated, none of the patients vomited after treatment and no severe adverse events were reported during the study. One non-pregnant woman had a PCR-confirmed new infection on day 35. None of the women presented recrudescent malaria during the nine weeks of follow-up.

**Table 1:** Admission demographic data of study population

	**Non-pregnant women**	**Pregnant women**	**P**-**value**
	**Median** (**Range**)	**Median** (**Range**)	
Number of patients	12	12	-
Daily piperaquine (phosphate) dose (mg/kg)	18.1 (15.1-24.2)	17.6 (13.6-21.7)	0.884
Daily piperaquine (base) dose (mg/kg)	10.5 (8.71-13.9)	10.2 (7.83-12.5)	0.884
Age (years)	21.0 (16.0-43.0)	26.0 (18.0-33.0)	0.977
Body weight (kg)	53.0 (44.0-81.0)	59.0 (50.0-72.0)	0.544
Height (cm)	163 (150–174)	166 (150–174)	0.908
Estimated gestational age (weeks)	-	32.0 (15.3–40.1)	-
Parasitemia (parasites/μL)	13200 (936–68700)	12900 (624–118000)	0.488
Days of fever	2.5 (1–6)	3 (1–6)	0.095
Fever (°C)	38.3 (36.7-40.0)	38.2 (36.6-39.9)	0.795
Haemoglobin (g/dL)	8.70 (7.60-11.5)	9.65 (8.00-12.0)	0.099
Urea (mg/dL)	26.0 (24.0-28.0)	25.0 (24.0-28.0)	0.036
Serum glutamic pyruvic transaminase (IU/L)	5.00 (2.00-10.0)	4.50 (2.00-9.00)	0.445
Serum glutamic oxaloacetic transaminase (IU/L)	13.5 (2.00-18.0)	13.0 (3.00-21.0)	0.727

The p-values are calculated using the Mann–Whitney U-test.

**Figure 1:**
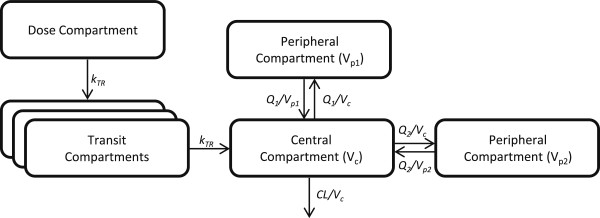
**The structural model for piperaquine pharmacokinetics.** k_tr_ is the 1^st^ order absorption rate. V_c_, V_p1_ and V_p2_ are the apparent volume of the central compartment, the first peripheral compartment and the second peripheral compartment, respectively. CL is the apparent elimination clearance and Q_1_ and Q_2_ is the apparent inter-compartmental clearances.

Five hundred sixty-four (564) post-dose plasma samples of piperaquine were used in the pharmacokinetic analysis. A total of 7 (1.2%) of these samples were measured to be below the limit of quantification and omitted. A three-compartmental disposition model resulted in a significantly better model fit compared with a two-compartment model (ΔOFV=−32.8). An additional peripheral compartment (four-compartment disposition model) did not further improve the fit (ΔOFV=0). A transit-compartment (n=3) absorption model was superior to all other absorption models (ΔOFV≥−44.5). In the final model the absorption rate constant (k_a_) and the transit-compartment rate constant (k_tr_) were set equal to increase the stability of the model. An additive residual error model was adequate in describing the residual random variability. BSV could be estimated reliably on elimination clearance, one inter-compartmental clearance parameter, and one peripheral volume parameter. The addition of BSV on the bioavailability resulted in a significant improvement in model fit (ΔOFV=−70.9). BOV had considerable impact on both mean transit absorption time and bioavailability (ΔOFV=−340). The final parameter estimates and a schematic picture of the final structural model is presented in Table 2 and Figure 1. Body weight was incorporated with an allometric function on all clearance and volume parameters (ΔOFV=−6.14).

**Table 2:** **Final parameter estimates describing the piperaquine population pharmacokinetics in women with uncomplicated *P*. *falciparum *malaria**

	**Population estimates [RSE %]**	**95% CI**	**BSV**/**BOV† [RSE %]**	**95% CI**
CL/F (L/h)	44.6 [9.90]	37.3-53.8	22.5 [31.5]	14.6-29.0
V_C_/F (L)	1820 [11.5]	1450-2240	-	-
Q_1_/F (L/h)	47.7 [19.0]	32.4-69.2	-	-
V_P1_/F (L)	15900 [12.3]	12600-20400	-	-
Q_2_/F (L/h)	352 [11.1]	283-431	-	-
V_P2_/F (L)	7520 [17.1]	5520-10500	-	-
MTT (h)	1.70 [8.05]	1.45-2.00	60.7 [22.8] †	44.5-76.7
RUV	0.0973 [5.90]	0.0753-0.120	-	-
No. of trans comp	3 *fix*	-	-	-
F (%)	100 *fix*	-	34.7 [59.2]	9.52-54.9
			64.8 [15.2] †	52.8-76.0

Coefficient of variation (%CV) for between-subject variability (BSV) and (†) between occasion variability (BOV) were calculated as the [exp(estimated variance)-1]^1/2^. Relative standard errors (RSE) and the 95% confidence intervals (CI) were based on 868 successful stratified bootstrap runs (out of 1000) and presented as 100×(standard deviation/mean value) and as 2.5 to 97.5 percentiles, respectively.

CL/F is the apparent elimination clearance. Vc/F is the apparent volume of distribution of the central compartment. Q1/F and Q2/F is the inter-compartment clearance between the central and first and second peripheral compartment, respectively. VP1/F and VP2/F is the apparent volume of distribution of first and second peripheral compartment, respectively. MTT is the mean transit time of the absorption model. RUV is the variance of the residual variability. No. of trans comp is the number of transit compartments used in the absorption phase. F represents the relative bioavailability.

Secondary parameters (i.e. total drug exposure, maximum concentration after first dose, time to maximum concentration, elimination half-life and day 7 concentrations) were analysed using the Mann–Whitney U-test to identify differences between pregnant and non-pregnant women (Table 3). There was a significant difference in terminal elimination half-life (p=0.0014), time to maximum concentration (p=0.0177) and maximum concentration (p=0.0205) [median (range) in pregnant vs. non-pregnant women: 22.1 (19.1-25.8) vs. 25.7 (20.9-33.3) days, 3.07 (1.65-4.64) vs. 1.48 (0.887-4.18) hours and 185 (109–363) vs. 102 (40.6-235) ng/mL, respectively]. However, no significant differences were found in day 7 concentrations (p=0.67), day 28 concentrations (p=0.84) or the total drug exposure (p=0.80) between the pregnant and non-pregnant women.

**Table 3:** **Secondary parameters of piperaquine pharmacokinetics in pregnant and non-pregnant women with uncomplicated *P*. *falciparum *malaria**

**Secondary parameters**	**Total**	**Non**-**pregnant women**	**Pregnant women**	**p**-**value**
C_MAX_ (ng/mL)	158 [40.6-363]	102 [40.6-235]	185 [109–363]	0.021
T_MAX_ (hours)	2.62 [0.887-4.64]	1.48 [0.887-4.18]	3.07 [1.65-4.64]	0.018
Half-life (days)	23.4 [19.1-33.3]	25.7 [20.9-33.3]	22.1 [19.1-25.8]	0.001
Day 7 concentration (ng/mL)	58.3 [16.6-146]	55.4 [16.6-146]	60.7 [40.1-103]	0.671
Day 28 concentration (ng/mL)	15.9 [4.85-38.6]	15.4 [4.85-38.6]	16.1 [9.68-26.8]	0.840
AUC_0->90_ (ng*h/mL)	40600 [12400–100000]	38000 [12400–100000]	42700 [27100–68700]	0.799
AUC_48h->90_ (ng*h/mL)	36400 [10600–90300]	35300 [10600–90300]	37700 [23500–63200]	0.887

Secondary parameters are predicted using the final model and values are presented as median [range]. The p-values are calculated with a Mann–Whitney U-test.

C_MAX_ is the predicted maximum concentration after the first dose and T_MAX_ is the time to C_MAX_. Half-life is the estimated terminal elimination half-life. Day 7 and 28 concentrations are the model predicted plasma concentrations of piperaquine at day 7 and 28, respectively. AUC_0->90_ is the predicted area under the concentration-time curve from time zero extrapolated to day 90. AUC_48h->90_ is the predicted area under the concentration-time curve from 48 hours extrapolated to day 90.

**Figure 2:**
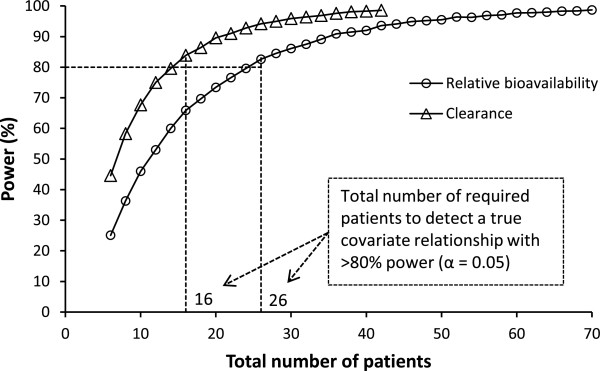
**Monte-Carlo Mapped Power (MCMP) curve for identifying pregnancy as a covariate.** Triangles represents the power curve for indentifying pregnancy as covariate on apparent elimination clearance and circles is the power curve for identifying pregnancy as a covariate on the relative bioavailability. The dotted black line represents 80% power. The inserted numbers are the total number of subjects needed to identify pregnancy as a covariate given the used model and study sampling procedure.

The MCMP analysis (5% significance level and 80% power) indicated that 8+8 and 13+13 women are needed to detect pregnancy as a covariate on elimination clearance and bioavailability, respectively (Figure 2). A formal stepwise covariate search was therefore not performed since it might result in a biased covariate selection in this small population sample.

**Figure 3:**
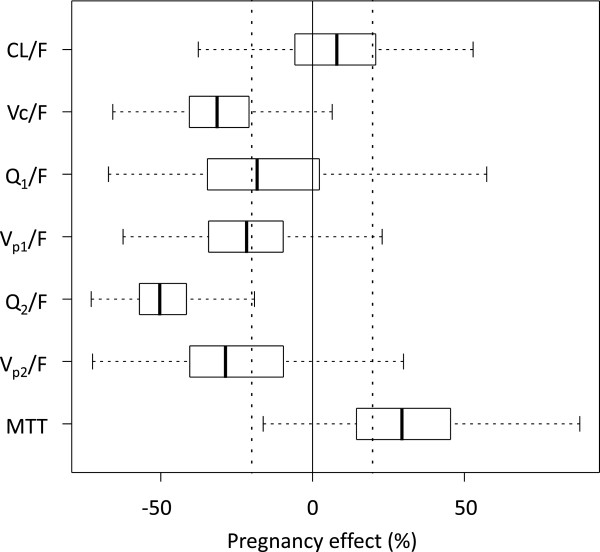
Box (25^th^ to 75^th^ percentile) and whisker (1.5*interquartile range) plot of the full pregnancy-covariate model for piperaquine. Pregnancy was included as a categorical covariate and the solid black zero-line represents no covariate effect and the dotted black lines represent a covariate effect of ±20%. MTT is the mean transit time, V_c_/F, V_p1_/F and V_p2_/F is the apparent volume of the central compartment, the first peripheral compartment and the second peripheral compartment respectively. CL/F is the apparent elimination clearance and Q_1_/F and Q_2_/F is the apparent inter-compartmental clearances.

**Figure 4:**
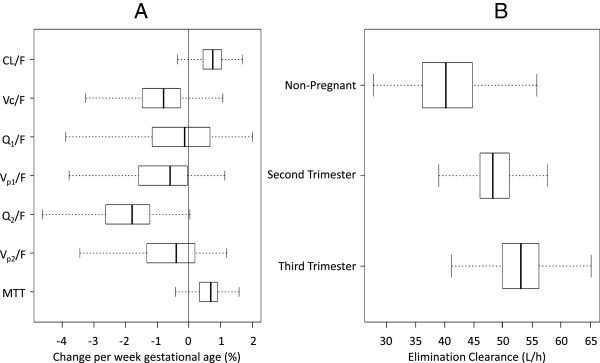
**Box** (**25^th^ to 75^th^ percentile**) and whisker (1.5*interquartile range) plot of the full covariate model with estimated gestational age implemented as a linear continuous covariate. Panel **A** displays the percent change per week of gestational age where the solid black zero-line represents no covariate effect. MTT is the mean transit time, V_c_/F, V_p1_/F and V_p2_/F is the apparent volume of the central compartment, the first peripheral compartment ant the second peripheral compartment, respectively. CL/F is the apparent elimination clearance and Q_1_/F and Q_2_/F is the apparent inter-compartmental clearances. Panel **B** displays the elimination clearance estimates for non-pregnant women, women in the second trimester (week 20) and women in the third trimester (week 32).

Two full covariate models were constructed from the final model to investigate the clinical relevance of pregnancy and EGA separately. Pregnancy had a relatively large impact on mean transit absorption-time, volume of distributions and inter-compartment clearances but no significant effect on elimination clearance (Figure 3). The inclusion of EGA as a power function or a linear function produced similar results. EGA was therefore implemented as a linear function for the full covariate approach and resulted in similar results compared to pregnancy as a categorical covariate (Figure 4A). Bootstrap results for elimination clearance, stratified by trimester (i.e. non-pregnant, second trimester at 20 weeks, and third trimester at 32 weeks) were also investigated and resulted in a non-significant trend of increasing clearance with increasing EGA (Figure 4B).

**Figure 5:**
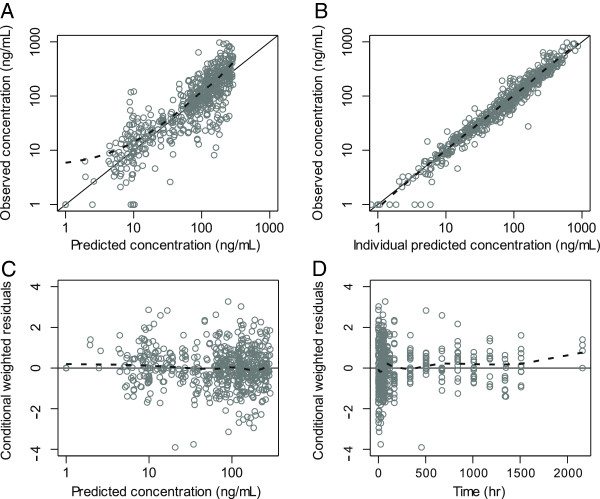
**Basic goodness-of-fit plots for the final piperaquine model.** Observations plotted against population predicted concentrations (**A**) and against individual predicted concentrations (**B**). Conditional weighted residuals plotted against population predicted concentrations (**C**) and time (**D**). The solid line is the identity line and the broken line is the locally weighted least square regression line. The concentrations were transformed into their logarithms (base 10). Conditional weighted residuals were fixed between 4 and −4, excluding one data point.

**Figure 6:**
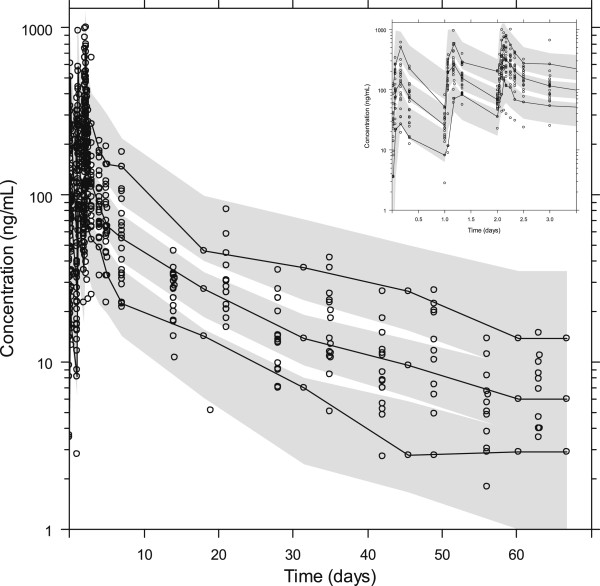
**The visual predictive check of the final piperaquine model.**. The circles represent the observations, the solid line represents the 5^th^, 50^th^ and 95^th^ percentile of the observed data. The shaded areas represent the simulated 95% confidence interval around the 5^th^, 50^th^ and 95^th^ percentile. Concentrations were transformed to their logarithms (base 10). Predicted performance during the three first days is inserted in the top right corner.

The final model resulted in good model diagnostic performance and reliable parameter estimates (Figure 5 and Table 2). Calculated epsilon-shrinkage was low (13.0%) which indicates that model diagnostics can be assessed reliably. However, eta-shrinkage was relatively high for certain parameters (CL/F=18.9%, MTT=13.2-52.8%, F=12.4-46.6%) (Table 2) and empirical Bayes estimates should therefore be interpreted with caution (Table 3) [52]. The final model had good predictive performance (Figure 6) with 4.8% (95% CI. 1.4%-11%) of observed data below and 2.1% (95% CI. 1.4%-10%) of observed data above the simulated 90% prediction interval (the three observations at day 90 were excluded because too few patients were followed up to this time for reliable simulations).

## Discussion

In this study, the pharmacokinetic properties of piperaquine have been investigated using nonlinear mixed-effects modelling in pregnant and non-pregnant Sudanese women treated with piperaquine-dihydroartemisinin for uncomplicated *P*. *falciparum* malaria. Few studies have been performed to evaluate the effect of pregnancy on the population pharmacokinetics of piperaquine, and this is the first study conducted in an African population. The treatment was well-tolerated and none of the participating women had recrudescent malaria infections.

Previous studies of piperaquine pharmacokinetics have presented both two- and three-compartment disposition models depending on the amount of data included in the analysis [14,15,17,19,20,53]. A three-compartment disposition model described the piperaquine concentration-time data adequately in this study. This supports the general finding that a three-compartment disposition is more appropriate than a two-compartment disposition when modelling data from patients followed for a sufficient period of time.

The absorption phase was best described with a transit compartment model with three transit compartments including random effects on bioavailability (BSV and BOV) and mean transit absorption time (BOV). The transit-compartment model provides a more physiological representation of the absorption process compared to the absorption models used in previous studies (i.e. first-order absorption and parallel first-order absorption with lag time) [15,17]. Recently published studies have also implemented the transit-compartment model but with two- and five-transit compartments which support the absorption model presented here [19,20]. Small variations in the number of transit compartments are to be expected when modelling different studies due to population differences, sampling schedules and study size. The inclusion of BSV and BOV in the absorption model improved the description of the data in the absorption phase and accommodated the large between-subject and between-occasion variability in these data. The data in the absorption phase was not rich enough to estimate separate absorption rates for k_a_ and k_tr_, and they were therefore set to be equal. This is a common limitation and the same approach has been used in previous studies [19,20]. Incorporating BOV resulted in an increasing median bioavailability (0.77, 1.19 and 1.40 at dose 1, 2 and 3, respectively) and mean transit absorption time (1.55, 1.95 and 2.05 at dose 1, 2 and 3, respectively) during the treatment regimen. Similar patterns have been identified in previously studies on piperaquine [19]. This might be an effect of disease recovery or differences in the food intake over the course of the dose regimen. This cannot be verified since parasite densities were not counted at each dose and food intake was not monitored in this study.

Pregnant women had a shorter terminal half-life compared to non-pregnant women, which is in agreement with the non-compartmental analysis [36], and higher maximum concentrations after the first dose. However, there were no differences in total piperaquine exposure, day 7 concentrations or day 28 concentrations, which supports previously published findings in an Asian pregnant population [19].

The main aim of this study was to investigate the pharmacokinetic differences between pregnant and non-pregnant women, but the sample size was not large enough to make a conventional covariate search. The MCMP analysis resulted in a minimum of 13 patients needed in each group in order to identify the previously described covariate relationships with 80% power and a significance level of 0.05 (Figure 2). However, this is under the assumption of perfect sampling since the MCMP analysis was based on simulated data using protocol sampling times. In the present study, some patients were not sampled for the complete follow-period and some samples were randomly missing which might increase the number of patients needed to identify the assumed covariate-relationships. Therefore the final model did not include any covariates except body weight, which has a strong biological prior [47,48] and in addition gave a drop in OFV when included in the model. The full covariate approach suffered from identifiability issues when incorporating the pregnancy covariate simultaneously on clearance parameters, volume parameters and relative bioavailability. The full covariate approach was therefore used to investigate the net effect of a potential covariate on all parameters except relative bioavailability. This approach resulted in a model with reduced volume of distribution and inter-compartment clearance in pregnant women compared with non-pregnant women but no net-effect on apparent clearance. This is in agreement with previously published results where pregnancy affected both clearance and relative bioavailability but in different directions [19]. These covariate relationships would also explain the difference in terminal elimination half-life and the lack of difference in total drug exposure.

The model presented in this study was built on data from few patients and a single individual can therefore have a considerable impact on the results. Piperaquine population pharmacokinetic parameter estimates from the final model are in agreement with previous reports (Table 4). However, the elimination clearance presented in this study for an African population is lower compared to previous studies in non-pregnant and pregnant patients. This might suggest an ethnicity related effect on elimination clearance but this needs to be confirmed in a larger population.

**Table 4:** A literature comparison of the pharmacokinetic properties of piperaquine

**Study population**	**Age** (**years**)	**Study size** (**Males**/**Females**)	**t**_**1**/**2**_ (**days**)	**CL**/**F** (**L**/**h**/**kg**)	**V**_**D**_/**F** (**L**/**kg**)	**Method**	**Reference**
**Piperaquine pharmacokinetics in pregnant women**							
Pregnant Sudanese women with uncomplicated *P*. *falciparum* malaria	18-33	12 (0/12)	22.1	0.678	384	Pop PK	This study
Pregnant Thai and Karen women with uncomplicated *P*. *falciparum* malaria	18-43	24 (0/24)	17.5	1.28	529	Pop PK	[19]
**Piperaquine pharmacokinetics in non**-**pregnant populations**							
Non-pregnant Sudanese women with uncomplicated *P*. *falciparum* malaria	16-43	12 (0/12)	25.7	0.739	446	Pop PK	This study
Non-pregnant Thai and Karen women with uncomplicated *P*. *falciparum* malaria	18-45	24 (0/24)	24.0	1.32	829	Pop PK	[19]
Non-pregnant Thai and Karen males and females with uncomplicated *P*. *falciparum* malaria	6-52	98 (59/39)	27.8	1.37	874	Pop PK	[17]
Non-pregnant Cambodian males and females with uncomplicated *P*. *falciparum* malaria	30±13†	38 (20/18)	22.6	0.900	574	Pop PK	[14]
Healthy Vietnamese males	21-45	12 (12/0)	23.0	1.82	194	Pop PK	[15]
**Piperaquine pharmacokinetics in children**							
Children in Papua New Guinea with uncomplicated *P*. *falciparum and P*. *vivax* malaria	7.1 ±1.5†	12 (8/4)	21.3	0.573*	385*	Pop PK	[54]
Children in Burkina Faso with uncomplicated *P*. *falciparum* malaria	2-10	236 (131/105)	23.2	0.417	214	Pop PK	[20]
Children in Papua New Guinea with uncomplicated *P*. *falciparum*, *P*. *vivax and P*. *malariae* malaria	6.9 ±1.4†	22 (17/5)	17.2	0.850	431	Pop PK	[53]
Cambodian children with uncomplicated *P*. *falciparum* malaria	7±2†	47 (26/21)	13.5	1.85	614	Pop PK	[14]
**Influence of diet on piperaquine pharmacokinetics**							
Fasting non-pregnant Thai and Karen males and females with uncomplicated *P*. *falciparum* malaria	18-55	15 (13/2)	17.5	1.19	700	NCA	[18]
Fed non-pregnant Thai and Karen males and females with uncomplicated *P*. *falciparum* malaria	19-41	15 (13/2)	21.4	1.01	769	NCA	[18]
Healthy Caucasian males and non-pregnant women, low fat meal	19-42	8 (4/4)	20.3*	1.14*	716*	NCA	[16]
Healthy Caucasian males and non-pregnant women, high fat meal	19-42	8 (4/4)	20.9*	0.60*	365*	NCA	[16]

CL/F is the apparent elimination clearance, t_1/2_ is the elimination half-life, V_D_/F is the apparent volume of distribution, Pop PK represents a population pharmacokinetic analysis and NCA represents a non-compartmental analysis. Age is given as a range or (†) as mean ± standard deviation. Other parameters are given as median or as mean when indicated (*).

In conclusion, this study presents the population pharmacokinetic properties of piperaquine in pregnant and non-pregnant women with uncomplicated *P*. *falciparum* malaria in Sudan. The terminal half-life was shorter in pregnant compared to non-pregnant women, but the total drug exposure was comparable between the two groups. This supports previous findings that no dose adjustments are needed on account of altered piperaquine pharmacokinetics in pregnancy.

## Competing interests

The Welcome Trust is a UK-based medical research charity and is independent of all drug companies. It has no financial links with the manufacturers of either the diagnostics tests or the drugs used in this study. The authors declare no conflict of interest.

## Authors’ contributions

IA, FN, ND, and NW conceived the project. WH, NL and JT quantified the drug concentrations. RH and JT performed the pharmacokinetic analysis and wrote the first draft of the manuscript. All authors revised the manuscript critically for important intellectual content and approved the final manuscript.
